# Supplementary material for: Natural selection drives chemical resistance of Datura stramonium
Source: PeerJ. 2016 Apr 14;4:e1898. doi: 10.7717/peerj.1898 (PMC4841232; doi:10.7717/peerj.1898)
Supplement: Table S1 — The number of each population corresponds to a locality given in Fig. 1. [file peerj-04-1898-s001.docx]

| No. | Population | N | Lang index | Altitude (m a.s.l.) | Latitude | Longitude | Mean Fruit production (*S.E.*) | Proportion of infested fruits (*S.E.*) | Infestation intensity (*S.E.*) | Remaining Sound Seeds (*S.E.*) |
| --- | --- | --- | --- | --- | --- | --- | --- | --- | --- | --- |
| 1 | Acatzingo | 30 | 40.48 | 2149 | 18.98 | -97.78 | 5.4(0.75) | 0.53(0.06) | 1.77(0.36) | 1265.34(233.12) |
| 2 | Actopan | 30 | 48.97 | 2008 | 20.26 | -98.93 | 2.9(0.31) | 0.74(0.07) | 2.20(0.33) | 83.1(25.71) |
| 3 | Ajacuba | 30 | 28.85 | 2222 | 20.08 | -99.11 | 3.16(0.57) | 0.67(0.07) | 3.17(0.45) | 127.46(39.2) |
| 4 | Atlixco | 30 | 48.97 | 1862 | 18.9 | -98.44 | 4.26(0.52) | 0.78(0.06) | 3.94(0.48) | 111.3(34.75) |
| 5 | Ciudad Hidalgo | 30 | 49.82 | 2060 | 19.69 | -100.55 | 4.6(0.48) | 0.42(0.07) | 0.56(0.11) | 454.96(136.53) |
| 6 | Coatepec | 18 | 101.36 | 1200 | 19.45 | -96.95 | 2.27(0.51) | 0(0) | 0(0) | 444.22(155.36) |
| 7 | Esperanza | 30 | 59.77 | 2460 | 18.86 | -97.37 | 3.93(0.38) | 0.74(0.07) | 5.07(0.68) | 292.13(64.9) |
| 8 | Huitzuco | 17 | 69.47 | 942 | 18.3 | -99.33 | 3.47(0.43) | 0(0) | 0(0) | 1239.94(181.7) |
| 9 | Iguala | 30 | 36.66 | 743 | 18.34 | -99.53 | 3.9(0.45) | 0.01(0) | 0.01(0.01) | 888.66(157.61) |
| 10 | Ixmiquilpan | 23 | 19.66 | 1724 | 20.48 | -99.21 | 3.34(0.63) | 0.57(0.09) | 2.03(0.45) | 112.95(32.98) |
| 11 | Jalancingo | 30 | 146 | 1904 | 19.81 | -97.33 | 3.96(0.36) | 0.01(0.01) | 0.26(0.26) | 1129.83(160.34) |
| 12 | Jalapa | 30 | 83.83 | 1429 | 19.54 | -96.91 | 2.6(0.24) | 0(0) | 0(0) | 487.46(88.43) |
| 13 | Janitzio | 29 | 61.1 | 2106 | 19.57 | -101.65 | 2.68(0.36) | 0.31(0.08) | 0.45(0.12) | 335.57(78.23) |
| 14 | Morelia | 30 | 44.33 | 1908 | 19.7 | -101.19 | 4.8(0.54) | 0.52(0.06) | 1.73(0.29) | 793.76(119.79) |
| 15 | Moroleon | 30 | 38.92 | 1817 | 20.12 | -101.19 | 6(0.65) | 0.33(0.07) | 1.45(0.38) | 1366.65(213.2) |
| 16 | Omitlan | 30 | 67.85 | 2432 | 20.16 | -98.65 | 4.66(0.89) | 0.81(0.06) | 2.63(0.34) | 179(43.88) |
| 17 | Patria Nueva | 30 | 34.37 | 1943 | 20.37 | -99.05 | 2.73(0.52) | 0.58(0.08) | 2.07(0.41) | 223.48(92.32) |
| 18 | Pedregal | 29 | 55.17 | 2314 | 19.31 | -99.18 | 3.66(0.61) | 0.3(0.07) | 0.87(0.27) | 440.36(158.1) |
| 19 | Perote | 30 | 41.38 | 2421 | 19.56 | -97.24 | 2.63(0.37) | 0.63(0.08) | 2.89(0.54) | 297.76(107.71) |
| 20 | Polotitlan | 7 | 46.42 | 2323 | 20.22 | -99.81 | 4.57(1.21) | 0.68(0.16) | 3.92(1.46) | 643.71(411.54) |
| 21 | Taxco | 30 | 66.37 | 1774 | 18.55 | -99.6 | 6.96(0.56) | 0.15(0.03) | 0.18(0.04) | 1676.33(165.88) |
| 22 | Teotihuacan | 30 | 37.8 | 2287 | 19.68 | -98.86 | 6.9(0.84) | 0.91(0.02) | 3.60(0.2) | 217.13(46.06) |
| 23 | Ticuman | 29 | 35.74 | 973 | 18.76 | -99.12 | 3.72(0.32) | 0.14(0.04) | 0.20(0.08) | 910.75(101.92) |
| 24 | Tixtla | 29 | 32.9 | 1360 | 17.56 | -99.39 | 6.13(0.73) | 0.6(0.05) | 1.27(0.22) | 1252.32(193.32) |
| 25 | Tlaxiaca | 21 | 44.66 | 2372 | 20.11 | -98.88 | 4.57(0.78) | 0.85(0.05) | 5.3(0.67) | 260.75(70.42) |
| 26 | Tula | 30 | 39.71 | 2171 | 20 | -99.22 | 2.7(0.27) | 0.78(0.06) | 4.20(0.61) | 170.63(64.16) |
| 27 | Tzin Tzun Tzan | 30 | 61.1 | 2054 | 19.63 | -101.57 | 4.63(0.89) | 0.21(0.06) | 0.78(0.28) | 333(75.61) |
| 28 | Valsequillo | 27 | 49.75 | 2064 | 18.9 | -98.18 | 6.66(0.96) | 0.63(0.08) | 3.66(0.56) | 437.38(68.49) |
| 29 | Xochipala | 30 | 75.01 | 1694 | 17.71 | -99.71 | 3.8(0.36) | 0.16(0.04) | 0.49(0.3) | 434.56(50.21) |
| 30 | Zacatlan | 30 | 73.43 | 2076 | 19.94 | -97.95 | 7.3(0.77) | 0.02(0.01) | 0.03(0.02) | 1459.36(199.17) |
| 31 | Zirahuen | 30 | 75.28 | 2174 | 19.43 | -101.91 | 3(0.38) | 0.02(0.01) | 0.02(0.01) | 663.4(99.44) |
