# Supplementary material for: Natural selection drives chemical resistance of Datura stramonium
Source: PeerJ. 2016 Apr 14;4:e1898. doi: 10.7717/peerj.1898 (PMC4841232; doi:10.7717/peerj.1898)
Supplement: Table S2 [file peerj-04-1898-s002.docx]

| No. | Population | Atropine mg•g^-1^ leaves | Scopolamine mg•g^-1^ leaves | Atropine mg•g^-1^ seeds | Scopolamine mg•g^-1^ seeds |
| --- | --- | --- | --- | --- | --- |
| 1 | Acatzingo | 0.1706 | 0.1333 | 0.1330 | 0.1415 |
| 2 | Actopan | 0.2999 | 0.4040 | 0.4869 | 0.6091 |
| 3 | Ajacuba | 0.0606 | 0.1895 | 0.1301 | 0.2495 |
| 4 | Atlixco | 0.6912 | 0.5775 | 0.1800 | 0.1956 |
| 5 | Ciudad Hidalgo | 0.4137 | 1.3528 | 0.1343 | 0.2237 |
| 6 | Coatepec | 0.0663 | 0.4225 |  |  |
| 7 | Esperanza | 0.5492 | 0.3935 | 0.2771 | 0.3633 |
| 8 | Huitzuco | 0.9018 | 0.3236 |  |  |
| 9 | Iguala | 2.7340 | 0.1598 |  |  |
| 10 | Ixmiquilpan | 0.6178 | 1.7547 |  |  |
| 11 | Jalancingo | 0.3469 | 0.5218 |  |  |
| 12 | Jalapa | 2.6889 | 2.5132 |  |  |
| 13 | Janitzio | 1.7500 | 2.4909 | 0.3613 | 0.1722 |
| 14 | Morelia | 0.6320 | 0.8962 | 0.1593 | 0.1672 |
| 15 | Moroleon | 0.5925 | 0.1827 | 0.1188 | 0.0995 |
| 16 | Omitlan | 0.2500 | 1.8436 |  |  |
| 17 | Patria Nueva | 0.2404 | 0.3781 |  |  |
| 18 | Pedregal | 0.6108 | 1.4523 |  |  |
| 19 | Perote | 0.0333 | 4.1006 |  |  |
| 20 | Polotitlan | 0.2538 | 0.0962 |  |  |
| 21 | Taxco | 0.4471 | 0.1903 | 0.2533 | 0.4170 |
| 22 | Teotihuacan | 0.4372 | 0.3538 |  |  |
| 23 | Ticuman | 0.9389 | 1.8890 |  |  |
| 24 | Tixtla | 0.4255 | 0.3067 |  |  |
| 25 | Tlaxiaca | 0.2888 | 0.4581 | 0.2275 | 0.3503 |
| 26 | Tula | 2.8345 | 2.0933 |  |  |
| 27 | Tzin Tzun Tzan | 0.9944 | 2.9955 | 0.1074 | 0.1664 |
| 28 | Valsequillo | 0.0275 | 1.5287 |  |  |
| 29 | Xochipala | 2.7530 | 0.2576 | 0.6278 | 0.3103 |
| 30 | Zacatlan | 3.0296 | 0.4494 |  |  |
| 31 | Zirahuen | 0.6185 | 1.9684 | 0.3825 | 0.2762 |
